# Fully Covered Metallic Stents for the Treatment of Benign Airway Stenosis

**DOI:** 10.1155/2016/8085216

**Published:** 2016-09-29

**Authors:** Caroline Dahlqvist, Sebahat Ocak, Maximilien Gourdin, Anne Sophie Dincq, Laurie Putz, Jean-Paul d'Odémont

**Affiliations:** ^1^Division of Pulmonology, CHU UCL Namur (Godinne Site), Université catholique de Louvain (UcL), Yvoir, Namur, Belgium; ^2^Department of Anaesthesiology, CHU UCL Namur (Godinne Site), Université catholique de Louvain (UcL), Yvoir, Namur, Belgium

## Abstract

*Introduction.* We herein report our experience with new fully covered self-expanding metallic stents in the setting of inoperable recurrent benign tracheobronchial stenosis.* Methods.* Between May 2010 and July 2014, 21 Micro-Tech® FC-SEMS (Nanjing Co., Republic of Korea) were placed in our hospital in 16 patients for inoperable, recurrent (after dilatation), and symptomatic benign airway stenosis. Their medical files were retrospectively reviewed in December 2014, with focus on stent's tolerance and durability data.* Results.* Twenty-one stents were inserted: 13 for posttransplant left main bronchus anastomotic stricture, seven for postintubation tracheal stenosis, and one for postlobectomy anastomotic stricture. Positioning was easy for all of them. Stents were in place for a mean duration of 282 days. The most common complications were granulation tissue development (35%), migration (30%), and sputum retention (15%). Fifty-five % of the stents (11/20) had to be removed because of various complications, without difficulty for all of them. None of the patients had life-threatening complications. * Conclusion.* Micro-Tech FC-SEMS were easy to position and to remove. While the rate of complications requiring stent removal was significant, no life-threatening complication occurred. Further studies are needed to better define their efficacy and safety in the treatment of benign airway disease.

## 1. Introduction

If surgery is usually considered as the first option for the treatment of a complex or recurrent benign tracheal stenosis [1], it is not always applicable because of the stenosis' extension and/or important comorbidities. In these cases, the use of an airway stent can offer good palliation. Unfortunately, none of the currently available stents are ideal. Silicone stents require a rigid bronchoscopy procedure for positioning and can have complications such as migration, development of granulation tissue, or sputum retention [2, 3]. Self-expandable metallic stents are easy to position (even with a flexible bronchoscope) and are less prone to migrate. However, they can also be obstructed by secretions, tumor overgrowth, or granulation tissue [4, 5]. Stent fractures have also been described [6]. When required, the removal of these metallic stents can be difficult [7, 8]. In 2005, in view of the high number of complications reported with metallic stents, the Food and Drug Administration (FDA) published an advisory on their use in benign airway lesions [9, 10].

Fully covered self-expandable metallic stents (FC-SEMS) have been developed in an effort to combine the advantages of metallic self-expanding stents with those of complete polymer coverage. There are only limited data on the use of FC-SEMS for the treatment of airway disease [11–16]. Widely different results have been published, which made it impossible to draw any definitive conclusion about this new variety of metallic stents, especially about their safety. The aim of this retrospective study is to assess the tolerance and the durability of Micro-Tech FC-SEMS (Nanjing Co., Korea) for the treatment of inoperable and recurrent symptomatic tracheobronchial benign stenosis.

## 2. Methods

Medical charts of all patients who underwent a Micro-Tech FC-SEMS placement for benign conditions between May 2010 and July 2014 were retrospectively reviewed in December 2014. Local institutional review board approved this study.

Collected data included patient's age and sex, type and location of airway disease, previous interventional bronchoscopic procedures (including or not including airway stenting), and current FC-SEMS outcome (impact on patient's symptoms, complications, and removal rate). Airway lesions were evaluated with computed tomography and/or bronchoscopy prior to stent placement. The central airway stenosis classification proposed by Freitag et al. was used to describe airway stenosis [17].

Micro-Tech FC-SEMS were used as a first choice for all the patients who required stenting for recurrent symptomatic inoperable benign airway stenosis during the previously described period after at least one unsuccessful airway dilatation and if their use was compatible with the bronchial anatomy. They were for instance not systematically used in the right bronchial tree as the absence of fenestration might have induced undesirable obstruction of the right upper bronchus. All the patients gave their agreement for the procedure.

**Figure 1:**
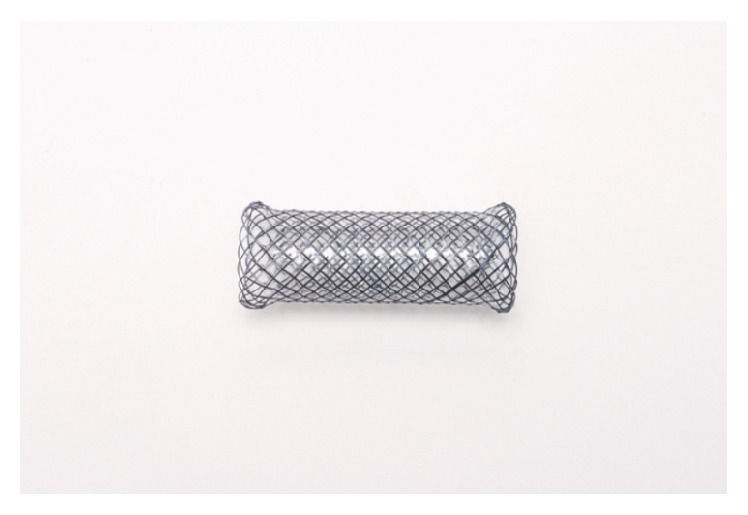
Micro-Tech straight fully covered self-expandable metallic stent (FC-SEMS).

Micro-Tech FC-SEMS (Nanjing Co., Korea) were made with a nitinol (titanium/nickel alloy) mesh completely covered internally with a silicone membrane. All the stents used in our study were straight stents (Figure 1), presenting a length of 30 to 60 mm and a diameter of 12 to 18 mm (with an additional 2 mm diameter at both extremities on a 2.5 mm length). All the stents were presented with a specific system produced by the same manufacturer for their deployment.

The stents were placed by our team of three pulmonologists specialized in interventional pulmonology (C.D., S.O., and J.-P. d'O.) during a rigid bronchoscopy procedure, under general anesthesia and with a fluoroscopic control. Ventilation was assured most of the time by high frequency jet ventilation or alternatively by conventional ventilation.

After stent placement, we defined three groups of complications: early (0–7 days), intermediate (7–90 days), and late (>90 days). In the absence of clinical symptoms, an endoscopic follow-up was scheduled every two weeks during the first six weeks after stenting. After that period, the follow-up was planned every two to three months or more often depending on the clinical symptoms.

Reintervention was defined as the necessity to perform, because of complications, a flexible bronchoscopy (e.g., fibroaspiration for mucous plugging or use of cryotherapy or argon plasma coagulation (APC) for tissue granulation), or a rigid bronchoscopy (e.g., stent removal). Stent removals were performed using the same type of procedure as for stent placement.

## 3. Results

Between May 2010 and July 2014, 21 straight Micro-Tech FC-SEMS stents were placed in 16 patients suffering from symptomatic benign airway stenosis.

Patient and stenosis characteristics are summarized in Table 1. Mean age was 55 years (range: 31–77). Seven patients were male and nine were female. All the patients with tracheal stenosis were judged to be inoperable because of the stenosis' length and/or comorbidities (mainly cardiological and respiratory). For transplantation patients, stent placement was always decided during a multidisciplinary discussion. All the patients had undergone at least one previous mechanical dilatation of their stenosis. Four patients (three with tracheal stenosis and one with posttransplant anastomotic stenosis) had previously been treated with a bronchial stent (silicone or partially covered SEMS) for the same airway disease but it had to be removed due to complications (e.g., migration and development of granulation tissue) or relapse of the airway stenosis.

Twenty-one straight FC-SEMS were inserted: thirteen for posttransplant left main bronchus anastomotic stricture, seven for postintubation tracheal stenosis, and one for a postlobectomy anastomotic stricture of the right bronchus intermedius.

Two stents were improperly positioned during the initial placement procedure (patients 8 and 9). The first one (patient 8) was partially repositioned during this initial procedure. However, it had to be removed 63 days later as it did not cover the stenosis anymore because of a secondary partial migration and the development of granulation. The second one (patient 9) had to be removed immediately because it was impossible to reposition it properly once deployed.

Stent placement provided symptom relief in all the patients. However, since this is a retrospective study, the clinical improvement was not scaled. Stents were in loco for a mean period of 282 days (range: 0–1618). No fatal adverse event occurred during or after the procedures. Complications are listed in Table 2. The most frequent ones were granulation tissue development (35%), migration (30%), and sputum retention (15%). Fifty-five % (11/20) of the stents had to be removed because of complications. All removal procedures were performed safely. The most frequent reason for removal was migration (5/11, 45%), which occurred 1 to 119 days (mean: 35 days) after stent placement. In 4/5 cases, the stent migrated within 10 mm of its original position, while in one (patient 8) it migrated 20 mm proximally. Two straight stents were successively removed in one patient (patient 2) because of the development of intrastent granulation tissue. Of note, these stents were placed on the top of a ruptured uncovered metallic stent. Patient 8 developed both migration and granulation in a context of improper placement as described earlier. Sputum retention was found during a routine endoscopy and treated with simple suction.

In one of the patients (patient 12), the stent had to be replaced because of its partial inefficiency in preventing bronchial closure in a context of severe posttransplant tracheobronchomalacia. A more proximal placement assured better functioning of the second stent.

The stents were all easily removed during a rigid bronchoscopy procedure. No mucosal tear or bleeding was observed after removal. All the retrieved stents were examined. Minor degradation of the silicone membrane was found in one case possibly due to the stent removal procedure. In the stents placed on the top of a ruptured uncovered metallic stent, a small disruption of the silicone membrane was found exactly where the granulomas were located.

## 4. Discussion

To date there are limited data on the use of FC-SEMS in airway disease and, to the best of our knowledge, no previous report on the use of Micro-Tech FC-SEMS in benign airway stenosis. In Table 3, we have summarized the main results from the four largest studies published on the topic, with a particular interest for those that included a large number of patients with benign airway stenosis [11–15].

These studies had very unequal conclusions: some authors considered FC-SEMS as dangerous [11] while others presented them as a safe and efficient treatment for airway obstruction [12–15]. The relatively small number of patients and the heterogeneity of airway disease and stent types probably contributed to these differences.

Stents are not the first option to consider for the treatment of benign airway stenosis. In benign tracheal stenosis, the treatment algorithm is well defined and surgery is considered as the treatment of choice. When surgery is not possible, mechanical dilatation is usually proposed and stents are placed only when dilatation fails to offer an acceptable result [18, 19]. In these cases, silicone stents are often considered as a better option, essentially because of their retrievable character. However, in posttransplant anastomotic strictures, it is not clear which treatment is the best as the stenosis can associate different characteristics (e.g., stenosis and malacia) or be concomitant with other airway complications (e.g., dehiscence or infections). Therefore, the treatment of a posttransplant anastomotic stenosis (surgery, mechanical dilatation, or stent placement) should always be determined on an individual basis during a multidisciplinary discussion [20]. When an airway stent is proposed for the treatment of a benign airway stenosis, the safety of the device is of capital importance. It should not break nor induce life-threatening complications. It should also be easy to place and to remove. Uncovered and partially covered metallic stents have been involved in so many complications that a FDA advisory against their use in benign stenosis has been published in 2005 [10].

In our study, we did not encounter any complication due to a stent quality defect (e.g., fracture or shriveling). The placement was easy and possible even in distal (e.g., close to the emergence of lobar bronchi) or tortuous lesions where it would have been difficult to use a silicone stent. When necessary, all Micro-Tech FC-SEMS were easily removed without complication, as opposed to what was reported with other metallic stents. The retrieved stents were examined and were found intact in all but one particular case of a patient with an underlying ruptured metallic stent.

Beside these qualities, we observed a significant rate of adverse events. Indeed, stent removal was required in >50% cases over a mean follow-up period of 282 days. However, this accounted for different situations in terms of severity. The most problematic issue was stent migration, which occurred in 30% cases and required the removal of five stents. Tracheal stenosis was involved in three out of the five cases of migration. For the stents located in the bronchus, migration was due to an initial mispositioning of the stent in patient 8 and very likely due to a too small stent diameter in patient 6. The migration rate found in our study but also in all previous publications with FC-SEMS [11–14] except one [15] was higher than in historic series with metallic stents [4]. The fully covered character of FC-SEMS and the absence of relief on their surface certainly lessen their adherence and probably facilitate their migration, especially in the trachea.

Granulation tissue development is a major issue with uncovered and partially covered SEMS (up to 57% cases in previous series [4]) and is one of the factors explaining their difficult removal. The fully covered membrane of the FC-SEMS was originally designed to decrease the development of extensive granulation. In our study, the rate of granulation tissue was significant (35%) but removal of the stent was never problematic. Indeed, the tissue extension was in general limited and located at the stent extremities. Granulation was found inside the stent in only one patient with an underlying broken metallic stent that had induced disruption of the silicone membrane.

Mucus plugging was found in 17% of our cases, all of them being asymptomatic. This is in line with previous studies with FC-SEMS (4.1 to 23.5% cases).

Our study included a large proportion of patients with a posttransplant anastomotic stricture of the left main bronchus. Metallic stents have previously been used to treat posttransplant complications but most of the available data were related to uncovered metallic stents. The most frequent issue with these uncovered or partially covered stents in posttransplant patients was the development of granulation tissue [4, 21, 22]. In 2008, Fernandez-Bussy et al. reported their experience with 49 AERO FC-SEMS in 24 patients with posttransplant airway disease (including bronchial stenosis, bronchomalacia, and dehiscence) [13]. Nineteen of these 49 stents (38%) had to be removed because of complications. Importantly, stent fracture was the cause for removal in three cases (6.1%). More recently, Tan et al. published their experience with six AERO FC-SEMS of small dimensions (diameter 10 to 12 mm, length 20 mm) in three patients with a posttransplant stenosis of the bronchus intermedius [16]. They found a very high migration rate (5/6) which seemed to be mainly linked to improper positioning or immediate migration. In this report, AERO stents did not seem to prevent the reoccurrence of bronchus intermedius stenosis either. Their removal was also problematic. In our view, FC-SEMS are probably not an adequate treatment for right posttransplant stricture because the segment to treat is too short and does not permit a correct anchorage of the stent, leading to a high probability of migration. In contrast, for posttransplant stenosis of the left main bronchus, we found Micro-Tech FC-SEMS to be an interesting alternative. In our experience, the positioning of the stent, even in tight and tortuous lesions, was easy (except in one case). The total migration rate in that particular group (2/8, 25%) was comparable to what has previously been reported by Fernandez-Bussy et al. (9/49, 18%) with AERO stents and by Dutau et al. with silicone stents (7/23; 30%) [23] in posttransplant patients, but the number of patients in our study was smaller.

Our study has several limitations. The main ones are related to its retrospective nature. First, while all our patients experienced disappearance or significant improvement of their symptoms (e.g., dyspnea, clinical signs of retrostenotic pneumoniae) after stent insertion, we were unable to document this reliably due to the retrospective nature of the file review. Second, the follow-up was unequal as some of the patients were referred to our tertiary clinic from distant hospitals and never came back after the stent placement. Third, patients with tracheal stenosis are underrepresented in our study because we are very reluctant to use metallic stents for the treatment of benign tracheal stenosis. This is in line with the FDA recommendations stating that metallic tracheal stents should be considered only after excluding other options in the hands of physicians experienced with these procedures. Finally, our sample size was small and therefore our observations should be validated in a larger cohort of patients.

In conclusion, we reported here a first experience with Micro-Tech FC-SEMS in benign airway stenosis. These stents were easy to position and to remove. Moreover, while the rate of complications requiring stent removal was significant, no life-threatening complication and no stent malfunctioning (e.g., fracturing and shriveling) occurred.

Despite the small sample size and the retrospective design of the study, we hypothesize that FC-SEMS may be a potential alternative for the treatment of recurrent symptomatic benign airway stenosis after a thorough exploration of the other therapeutic options in line with the FDA recommendations related to the use of SEMS. Patients with symptomatic, tight, and tortuous left posttransplant strictures to whom only few options apply may be the best candidates. Further prospective randomized controlled studies comparing different types of stents are needed to better define the efficacy and safety of FC-SEMS in the treatment of benign airway stenosis.

## Figures and Tables

**Table 1:** Patients, stenosis, and stent characteristics.

Patients' characteristics			Stenosis characteristics					Stent characteristics		Number of previous stents
Number	Age	Sex	Diagnosis	Type	Degree	Location	Length	Type	Dimensions	
1	31	M	PITS	S4-D1	4	II	30	Straight	16*∗*50	1
	34	M	PITS	S4-D1	4	II	30	Straight	16*∗*50	2

2	56	M	PITS	S4	4	II	15	Straight	14*∗*40	2
	56	M	PITS	S4	4	II	15	Straight	14*∗*40	3

3	56	M	PITS	S4	2	I	10	Straight	18*∗*50	0

4	54	F	PITS	S4	3	I	35	Straight	18*∗*60	1

5	59	F	AS-LTX	S4-D1	2	V	15	Straight	12*∗*40	0
	61	F	AS-LTX	S4-D1	4	V	15	Straight	12*∗*50	1

6	62	F	AS-LTX	S4	4	V	20	Straight	8*∗*40	0

7	51	M	AS-LTX	S4-D1	5	V	20	Straight	12*∗*40	0

8	77	F	AS-SL	S4	3	IV	20	Straight	12*∗*30	0

9	57	F	AS-LTX	D1	4	V	10	Straight	14*∗*40	0

10	61	F	AS-LTX	S4-D1	4	V	10	Straight	14*∗*40	0

11	54	F	AS-LTX	S4	3	V	15	Straight	14*∗*40	4

12	57	M	AS-LTX	D1	3	V	50	Straight	14*∗*40	0
	57	M	AS-LTX	D1	3	V	50	Straight	14*∗*40	1

13	58	M	AS-LTX	S4	4	V	10	Straight	12*∗*40	0
	58	M	AS-LTX	S4	4	V	25	Straight	12*∗*40	1

14	58	F	AS-LTX	D1	4	V	35	Straight	14*∗*40	0

15	49	M	PITS	S4-D1	3	I	30	Straight	20*∗*40	0

16	65	F	AS-LTX	S4	4	V	15	Straight	12*∗*40	0

M = male, F = female, PITS = postintubation tracheal stenosis, AS = anastomotic stricture, SL = sleeve lobectomy, LTX = lung transplantation.

**Table 2:** Complications.

Number	Disease	Stent					
		Follow up (days)	Complications			Reintervention other than removal	Cause for removal
			Early	Intermediate	Late		
1	PITS	1618			Halitosis		Halitosis
	PITS	428					Still in place

2	PITS	205		Granulation	Halitosis	APC on granulation tissue	Granulation
	PITS	441		Granulation	Halitosis		Granulation

3	PITS	258	Migration		Restenosis at upper extremity	Dilatation	Restenosis

4	PITS	21		Migration	NA		Migration

5	AS-LTX	924		Granulation		APC on granulation tissue	In place for 3 years
	AS-LTX	190					Still in place

6	AS-LTX	3	Migration	NA	NA		Migration

7	AS-LTX	126		Granulation, sputum retention		Fibroaspiration	During flexible bronchoscopy

8	AS-SL	63		Migration, granulation	NA		Migration

9	AS-LTX	0	Immediate removal	NA	NA		Immediate removal

10	AS-LTX	126		Sputum retention	Migration		Migration

11	AS-LTX	141		Restenosis at lower extremity		Dilatation	Still in place

12	AS-LTX	112					Partial inefficiency
	AS-LTX	463					Still in place

13	AS-LTX	203		Restenosis at lower extremity	NA		Restenosis
	AS-LTX	337					Still in place

14	AS-LTX	111	Sputum retention		Granulation	Fibroaspiration	Patient died of another cause

15	PITS	3	Migration	NA	NA		Migration

16	AS-LTX	155			Granulation		Still in place

PITS = postintubation tracheal stenosis, SL = sleeve lobectomy, LTX = lung transplantation, NA = not applicable, and APC = argon plasma coagulation.

**Table 3:** 

	Shin et al. [12]	Fernandez-Bussy et al. [13]	Dooms et al. [11]	Chen et al. [14]	Marchese et al. [15]	This publication
*Airway lesion characteristics*						
Benign/malignant	6/9	49//0	17/0	21/0	50/2	16/0
*Location *						
Trachea and carina	7	0	14	21	2	5
Right bronchus	0	20	1	0	14	1
Left bronchus	8	29	2	0	36	10

*Stent characteristics*						
Manufacturer	Taewoong	Alveolus	Silmet (*n* = 7)	Sigma CZTS	Silmet	Micro-Tech
			Taewoong (*n* = 8)			
			Alveolus (*n* = 5)			
Straight/Y/conical	**17/0/0**	**49/0/0**	**20/0/0**	**27/0/0**	**31/0/21**	**21/0/0**
Metal type	Nitinol	Nitinol	Nitinol	Stainless steel	Nitinol	Nitinol
Cover	PTFE	Silicone	*Silmet*: Silicone	Silastic	Silicone	Silicone
			*Taewoong*: PTFE			
			*Alveolus*: PU			

*Correct positioning *	100% (17/17)	100% (49/49)	100% (20/20)	100% (27/27)	98% (51/52)	90% (19/21)

*Follow-up*	270 days	7 months	12 weeks	5 months	119 days	282 days

*Complications *						
Overall complication rate	59% (10/17)	48% (24/49)	90% (18/20)	85% (23/27)	40% (20/52)	75% (15/20)
Requiring removal of stent	41% (7/17)	39% (19/49)	80% (16/20)	22% (6/27)	15% (7/52)	57% (12/21)
Most frequent complications	Sputum retention (23.5%) Stent migration (17.6%) Tissue hyperplasia (17.6%)	Granulation (20.4%) Migration (18.4%) Fracture (6.1%) Mucus plugging (4.1%)	Migration (60%) Wrinkling (10%) Stent fracture (5%)	Granulation (18/21 patients, 89%) Stent migration (6/27 stents, 22%) Halitosis (6/21 patients, 28%)	Tumor overgrowth (15%) Stent migration (13.4%) Granulation (3.8%) Infection (3%)	Granulation (35%) Migration (30%) Sputum retention (17%)

PTFE = polytetrafluoroethylene; PU = polyurethane.
