# Corrigendum to “Fully Covered Metallic Stents for the Treatment of Benign Airway Stenosis”

**DOI:** 10.1155/2018/6202750

**Published:** 2018-05-22

**Authors:** Caroline Dahlqvist, Sebahat Ocak, Maximilien Gourdin, Anne Sophie Dincq, Laurie Putz, Jean-Paul d'Odémont

**Affiliations:** ^1^Division of Pulmonology, CHU UCL Namur (Godinne Site), Université catholique de Louvain (UcL), Yvoir, Namur, Belgium; ^2^Department of Anaesthesiology, CHU UCL Namur (Godinne Site), Université catholique de Louvain (UcL), Yvoir, Namur, Belgium

In the article titled “Fully Covered Metallic Stents for the Treatment of Benign Airway Stenosis” [1], the location of the company “Micro-Tech® FC-SEMS” was incorrectly stated in the Abstract as “Micro-Tech® FC-SEMS (Nanjing Co., Republic of Korea)” and in the Introduction and Methods as “Micro-TechFC-SEMS (Nanjing Co., Korea).” It should be corrected to “Micro-Tech® FC-SEMS (Nanjing Co., China).
